# On the Semeiology of the Tongue

**Published:** 1849-01

**Authors:** Samuel Wright

**Affiliations:** Birmingham


### ARTICLE II.

*On the Semeiology of the Tongue.* By SAMUEL WRIGHT, M. D., of Birmingham.—(Clinical Lectures in Medical Times.)

Whilst some are disposed, in a prodigality of prejudice, to look upon the tongue as pathognomonic of nearly all the "ills that flesh is heir to," others make comparatively light of it, and consider its testimony as little trustworthy. To be amongst the best judges on the subject, is to belong to neither of those parties. As a rule, the tongue is a very faithful indication of the condition of the alimentary organs; but its evidences are not unexceptionable. A furred tongue, for instance, is a common indication of dyspepsia, but it is not a constant one. We sometimes meet with irritable nervous subjects, whose tongues are habitually furred, yet without any signs or symptoms whatever of gastric derangement. Others, again, will have clean tongues, and of natural redness, whilst they are suffering from severe stomach disorder. Various circumstances exert a remarkable influence upon this organ. Some people, otherwise healthy, get a furred, clammy tongue, if their stomachs are empty a little longer than usual. Others have their tongues always furred when their stomachs are full; the coating continues only during digestion, and passes off as this function ceases, Mental and moral emotions affect the condition of the tongue in a singular manner; perhaps it never becomes morbid without the nervous function, in its higher offices, being somewhat implicated. This would explain why a furred tongue is so rarely met with in the inferior animals. It may happen, and I think not unlikely, that in dyspepsia, the disorder the brain suffers, sympathetically with the stomach, has as much share as this organ itself in giving the tongue its characteristic coating. Certain it is, as I have said, that the feelings of the mind will, in a very few minutes, render a clean tongue a foul one. This is a subject which I have been induced curiously to inquire into for some years past, and I have seldom met with an exception to what I have just observed. Among the profoundly studious, amongst those terrified by sudden apprehensions, or shocked by the sudden advent of ill news; among the hypochondriacal, hysterical, gloomy, and desponding, you will find many examples of the mind's influence, in this particular, upon the body. A patient of mine, living near this town, will well illustrate what I say. He is a man of remarkably good constitution, and moulded like a miniature Hercules. Moreover, he has no incumbrances; an excellent mercantile business, that takes up little of his time, is partial employment for him, leaving him many leisure hours in every day that he has some difficulty in disposing of. This he chiefly occupies in fancying himself the victim of all possible kinds of ailments. There is no disease in the nosology too much for his imagination. Of course, these things are all imaginary, and tiresome enough to listen to, when your judgment and sense of justice tell you that it is not a case for "physic and a physician." You will anticipate my saying that this gentleman is possessed of a most unfortunate nervous sensibility, which chiefly manifests itself in an ideal pathology, all reflected upon his own person. The peculiarity in point, however, which I chiefly wish to speak of, refers to his tongue. I had never seen him with this organ quite clean (although I have not once attended him for dyspepsia), yet the readiness with which it acquires a fur is very remarkable. Many times have I examined his tongue, and found it comparatively what it ought to be, before hearing a recital of his imaginary maladies; and after this, in some quarter or half an hour's detail, that same tongue has put on an aspect almost like that of flannel. I am at this time attending with Mr. Carter, a patient, one amongst the pitiable many who have seen better days. I shall take occasion hereafter to give you his case in due detail, but for the present, I may observe that his tongue has the peculiarity characteristic of the one just spoken of. I should premise, however, that there is a fancied trouble in the one instance, and a matter-of-fact one in the other. Four days ago, in calling upon the gentleman I am now alluding to, one of the first things I did was to look at his tongue. I found it, as usual, very pale, flabby, and moist, but without any coating. After having made other necessary inquiries, I was informed by my patient that his heart, which has long been disturbed by mental emotion, the other night beat with unusual vehemence and irregularity. On my asking if he could account for it, he told me that he had just then received the distressing intelligence that an uncle, from whom he expected a competency, had not left him a shilling! This pitiable tale, told with much earnestness and visible feeling, occupied little more than twenty minutes; at the end of that time I again looked at his tongue, and *found it coated with a thick, white fur!*

I mention these things, thus generally, to you, not only as items in pathology with which you ought to be made familiar, but also as suggestive of a discreet rule of practice, viz.: to let the examination of a patient's tongue be *one of your first duties at his bedside.* My own experience, perhaps not inconsiderable on this point, enables me to say that in nine cases out of ten, and more especially among females, the tongue will be found, on first entering the room, in a very different state to what it is after half an hour's questioning and manipulation.—*South. Med. & Surg. Jour.*

